# Worker Survival and Egg Production—But Not Transcriptional Activity—Respond to Queen Number in the Highly Polygynous, Invasive Ant *Tapinoma magnum*

**DOI:** 10.1111/mec.17679

**Published:** 2025-02-04

**Authors:** Anna Lenhart, Megha Majoe, Sibel Selvi, Thomas J. Colgan, Romain Libbrecht, Susanne Foitzik

**Affiliations:** ^1^ Institute of Organismic and Molecular Evolution Johannes Gutenberg University Mainz Mainz Germany; ^2^ Department of Evolutionary Biology and Ecology Institute of Biology I (Zoology) Albert Ludwig University of Freiburg Freiburg Germany; ^3^ Insect Biology Research Institute UMR 7261, CNRS, University of Tours Tours France

**Keywords:** ageing, gene expression, invasive species, life history, polygyny, reproductive division of labour

## Abstract

In social animals, reproductive activity and ageing are influenced by group composition. In monogynous (single‐queen) insect societies, queen presence affects worker fecundity and longevity, but less is known about worker responses to queen number variation in polygynous (multi‐queen) species or how queens age in these systems. We created queenless, one‐queen and two‐queen colonies of the invasive, polygynous ant *Tapinoma magnum* to examine the effect of queen number on worker survival, ovary and oocyte development, oxidative stress resistance and fat body gene expression. We also compared the fecundity and brain and fat body transcriptomes between young and old queens. Queenless workers experienced the highest mortality, contrasting with monogynous species, where queen removal typically extends lifespan. Workers lived longer and had more developing oocytes in their ovaries in single‐queen than in two‐queen colonies. Queen number did not directly affect oxidative stress resistance or fat body gene expression, though its effect on the latter differed between inside and outside workers. Furthermore, inside—likely younger—workers produced more oocytes, showed higher oxidative stress resistance and upregulated antioxidant genes compared to outside—likely older—workers. Minimal shifts in fecundity and gene expression of differently aged queens indicated their physiological stability. Our research highlights distinct caste‐ and tissue‐specific responses to varying queen numbers in workers of a highly polygynous species.

## 1 Introduction

A fundamental tenet of life history theory predicts that organisms are confronted with trade‐offs. These trade‐offs can be driven by resource allocation, for example, organisms must decide whether to invest more resources into body maintenance or reproduction. They can also evolve as a consequence of suboptimal gene regulatory networks for both reproduction and body maintenance (Kirkwood 2017; Maklakov and Chapman 2019). Ageing—the inevitable intrinsic deterioration with age, limits the reproductive potential of an organism, often resulting in a negative correlation between longevity and fecundity (Chapuisat and Keller 2002; Partridge and Harvey 1988). As organisms age, the accumulation of molecular and cellular damage leads to functional decline and increased risk of disease and death with advancing age (Kirkwood et al. 1997). The manifestation of reactive oxygen species (ROS) and the organism's fight to neutralise its damage result in oxidative stress, a proximate cause of ageing. Metabolically costly activities such as reproduction may favour ROS production (Kramer et al. 2021; Selman et al. 2012). If organisms invest into antioxidants, ROS can be neutralised before critical damage occurs (Münch, Amdam, and Wolschin 2008; Ray, Huang, and Tsuji 2012; Seehuus et al. 2006).

Social insects (ants, termites and some bees and wasps) are excellent models to study the evolution of senescence. Obligate reproductive division of labour has led to distinct phenotypes with varying morphologies, behaviours, lifespans and fecundities. Queens are highly fertile and long‐lived, while workers are typically sterile and short‐lived (Keller 1998; Kramer, Schaible, and Scheuerlein 2016), representing a reversal of the typical trade‐off between longevity and fecundity. This may be attributed to the ample resources queens receive from their colonies (Hölldobler and Wilson 1990; Negroni, Foitzik, and Feldmeyer 2019; Kramer et al. 2022). Although the molecular mechanisms driving caste‐specific ageing are not fully resolved, queens are known to invest more into body repair and maintenance, contributing to their extended lifespans, which may last for decades in ants and termites (Lin, Werle, and Korb 2021; Negroni, Foitzik, and Feldmeyer 2019).

Queen lifespan is also influenced by environmental and social factors. In ants, the average life expectancy of a queen is much higher in species with single‐queen societies than in polygynous species with several queens per colony (Keller and Genoud 1997). Queen number in the colony is often linked to the colony founding strategy. In monogynous species, queens are well‐provisioned and typically found colonies independently (e.g., *Lasius niger* ; Janet 1907). In contrast, queens of polygynous species often mate locally and stay in their natal nest (e.g., *Formica selysi*; Avril et al. 2019, Keller 1995). Queen loss in monogynous societies ultimately leads to the demise of the colony and impacts worker behaviour and physiology. In polygynous societies, losing one queen has minor consequences as queens can be easily replaced.

Worker reproduction under queenright conditions is rare, likely due to the emission of pheromones by queens indicating their presence and fecundity (Holman 2018; Oliveira et al. 2020; Bourke 1988; Foitzik and Herbers 2001). Worker sterility is found in many invasive species and more generally in species with large colonies (Aron, Keller, and Passera 2001; Dijkstra and Boomsma 2006). Under queenless conditions, workers of many ants develop their ovaries, fight over reproductive dominance and lay haploid, male‐destined eggs (Boomsma 2009; Heinze, Puchinger, and Hölldobler 1997). The onset of egg production by queenless workers is typically associated with shifts in their physiology and immunity. These workers exhibit increased oxidative stress resistance, altered brain and fat body gene expression and frequently have extended lifespans compared to queenright workers (Hartmann and Heinze, 2003; Dixon, Kuster, and Rueppell 2014; Blacher, Huggins, and Bourke 2017; Kuszewska et al. 2017; Kohlmeier et al. 2017; Lopes, Campbell, and Contrera 2020; Majoe et al. 2021). Worker fecundity is also influenced by age and role within the colony (Bourke 1988). Younger workers care for brood, while older workers engage in riskier tasks like foraging and defence (Tofilski 2002; Wilson 1971, 1985). This age polyethism connects age, behaviour, fecundity and nest location, with younger inside workers more likely to activate their ovaries under queenless conditions (Giraldo and Traniello, 2014; Bourke 1988; Seistrup et al. 2023; Blacher, Huggins, and Bourke 2017).

In contrast to species where workers have functional ovaries, sterile workers cannot reproduce, even in queenless nests. Thus, they should not break the aforementioned longevity/ fecundity trade‐off. For instance, queen loss in the supercolonial ant *Lasius neglectus* did not affect the susceptibility to oxidative stress and gene expression in sterile workers (Majoe et al. 2024). In species with functionally sterile workers, workers may still have ovaries and lay nutrient‐rich, non‐viable trophic eggs that serve as high‐quality food for larvae but cannot develop into offspring. By using these eggs to rear the queen's offspring, workers can increase their indirect fitness but cannot achieve direct fitness benefits. Studying invasive, polygynous species with functionally sterile workers who are unlikely to experience queen loss in nature, may help determine if extended worker lifespans seen in species with reproductive workers also apply to functionally sterile workers with active ovaries that lay trophic eggs.

The dolichoderine ant *Tapinoma magnum* is an ideal system for studying invasive species dynamics. As part of the West and Central Mediterranean *Tapinoma nigerrimum* complex, it has the broadest distribution and invasive potential (Dekoninck, Parmentier, and Seifert 2016; Seifert et al. 2017). In its invasive range, *T. magnum* is highly polygynous and forms supercolonies with thousands of workers and up to 350 queens per nest spot. Most mated young queens remain near their natal colony and are adopted into neighbouring nests (Seifert et al. 2017). Workers display a size polymorphism (Dekoninck, Parmentier, and Seifert 2016; Seifert et al. 2017) and possess well‐developed ovaries (pers. observation); however, worker‐laid eggs appear to be non‐viable trophic eggs, as we did not observe them to develop beyond the egg stage.

Previous studies that investigated the relationship between longevity and fecundity in social insects primarily examined the effects of queen loss in monogynous or facultatively polygynous species, revealing a positive link between worker survival and fecundity, driven by enhanced investment in antioxidants and detoxification enzymes under queenless conditions (Majoe et al. 2021; Negroni et al. 2021). In this study, we extended this framework by examining the effects of queen loss, monogyny and polygyny on worker survival, ovarian development, oxidative stress resistance and fat body gene expression (linked to energy storage and reproduction; Arrese and Soulages 2010; Cervoni et al. 2017) in a strictly polygynous species. Additionally, we explored whether queen loss or queen number elicits similar physiological and molecular responses in workers (Kohlmeier et al. 2017; Negroni et al. 2021; Majoe et al. 2021). Since age‐related division of labour is often linked to physiological changes in ovary development and oxidative stress resistance (Hartmann and Heinze, 2003; Giraldo and Traniello, 2014; Quque et al. 2023), we hypothesized that worker location would correlate with age, ovary development, oxidative stress resistance, and fat body gene expression (Kramer et al. 2021; Majoe et al. 2021; Seistrup et al. 2023; Koto et al. 2023). We expected inside workers to exhibit stronger physiological responses to queen number than outside workers, as they interact more closely with the queen. Additionally, we predicted that inside workers would demonstrate greater resistances to oxidative stress, consistent with findings in other species (Majoe et al. 2021; Kramer et al. 2021; Kennedy, Herman, and Rueppell 2021).

Within a complementary experiment, we explored how queen age influences fecundity and gene expression. Age‐related changes in queens influence reproduction and molecular pathways (Negroni, Foitzik, and Feldmeyer 2019; Von Wyschetzki et al. 2015), while queen lifespan is often tied to social structure (Hölldobler and Wilson 1977; Keller and Genoud 1997; Kramer et al. 2022). Queens of the polygynous form of the red imported fire ant, *Solenopsis invicta* , are estimated to live between 1 and 3 years (Goodisman and Ross 1999), while queens of the similarly invasive dolichoderine ant, *Linepithema humile* , typically have lifespans of approximately 1 year (Reuter et al. 2001). Although the exact lifespan of *Tapinoma magnum* queens is unknown, our laboratory observations show that dealated queens can live well over 1 year. Based on these observations and the estimated lifespans of comparable invasive and polygynous species, we hypothesized that *T. magnum* queens may have average lifespans in the range of 1–3 years (Goodisman and Ross 1999; Reuter et al. 2001; Keller and Genoud 1997). In short‐lived *Cardiocondyla obscurior* queens, fecundity remains consistent until the end of life, whereas long‐lived *Temnothorax rugatulus* queens transition from prioritising immune function to antioxidant production as they age (Jaimes‐Nino, Heinze, and Oettler 2022; Negroni, Foitzik, and Feldmeyer 2019). Unlike long‐lived queens of monogynous ants that can survive for decades, shorter‐lived queens, such as those of *T. magnum* , may also exhibit divergent ageing trajectories. By analysing gene expression in the brain and fat body—key tissues for hormone production and protein synthesis (Corona et al. 2007; Min and Benzer 1997)—we sought to understand how ageing impacts queen physiology. This approach allowed us to compare age‐related transcriptional changes in queens and workers, offering a comprehensive perspective on the links between age, reproduction and molecular changes in *T. magnum* . Together, these experiments illuminate how social structure and ageing shape the physiological and molecular underpinnings of colony dynamics.

## 2 Material and Methods

### 2.1 Study Site, Collection and Laboratory Maintenance

We collected ants from a supercolony of *Tapinoma magnum* on a strip of fallow land, formerly a plant nursery, in Ingelheim am Rhein, Germany (49°58′39.8″N, 8°03′17.3″ E) in June and July 2020, and in June 2021. In 2020, 13 dealate queens, a few thousand workers and brood were collected. In 2021, we collected 13 alate queens, over 30 males, 50 dealate queens and a few thousand workers and brood from multiple nest chambers, each typically housing two to four queens. Colonies were maintained in a 25°C climate chamber at the Johannes Gutenberg University Mainz. The 13 dealate queens and workers from 2020 and the 50 dealate queens and workers from 2021 were housed in separate boxes (78 cm × 56 cm × 18 cm), coated with Fluon (Whitford GmbH, Diez, Germany) to prevent escapes. Each box contained three artificial nests, each constructed from two glued Petri dishes connected by a 1 cm hole plugged with cotton. The lower Petri dish (7.5 cm diameter, 4 cm high) served as a water reservoir, the upper Petri dish (9.5 cm diameter, 1.2 cm high) functioned as nest entrance and was covered with a loose lid containing small holes. Each nest was covered by a plastic flowerpot (13 cm diameter, 12 cm high) to create a dark environment. Colonies were fed twice weekly with a mix of honey, eggs and vitamins, and once weekly with crickets. Queens collected in 2020 were maintained under these conditions for at least 16 months, while alate queens from 2021 were housed in a separate box (78 cm × 56 cm × 18 cm) with males to ensure fertilisation. After copulation, the young queens shed their wings and became reproductive.

### 2.2 Experimental Set‐Up

**FIGURE 1:**
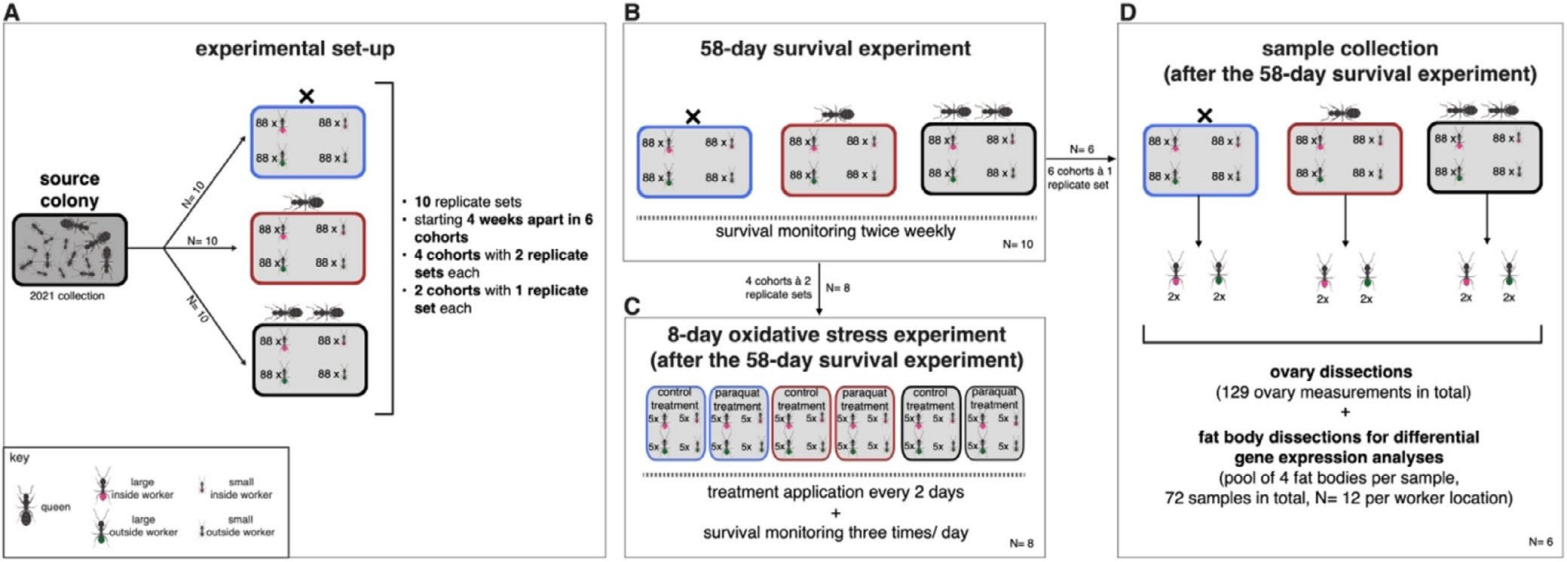
Experimental set‐up. (A) The source colony was divided into 10 replicate sets, each containing three sub‐colonies with no queen (queenless treatment, blue box, *N* = 10), one queen (monogynous treatment, red box, *N* = 10) and two queens (polygynous treatment, grey box, *N* = 10). Each sub‐colony consisted of 352 workers comprising of similar numbers of large/small inside and outside workers. The 10 replicate sets started at six time‐points (cohorts) 4 weeks apart (four cohorts à two replicate sets and two cohorts à one replicate set). (B) Worker mortality was documented twice per week for 58 days. (C) For the oxidative stress experiment, 10 large and 10 small inside workers, as well as 10 large and 10 small outside workers from each treatment (*N* = 8) were either subjected to an oxidative stress treatment (Paraquat dichloride solution) or control treatment (Millipore water) for 8 days. Treatments were repeated every 2 days and survival was monitored three times daily. (D) For the ovary dissections and the differential expression analyses, large inside (pink, *N* = 12) and outside (green, *N* = 12) workers from the three treatments were used. We used two samples per worker location across six replicate sets from six cohorts (*N* = 6). Each fat body sample (72 samples) was a pool of four individual fat bodies used for differential expression analysis. Ovary and fat body dissections were conducted simultaneously. We measured 129 worker ovaries (out of 144 dissected workers).

To investigate the effect of queen number on worker survival, ovarian development and gene expression, we divided the source colony collected in 2021 into 10 replicate sets, each containing three sub‐colonies housed in separate boxes (40 cm × 33.5 cm × 17 cm) with one nest covered by a plastic flowerpot (as described above). Each sub‐colony was randomly assigned to one of three treatments: ‘queenless’ (no queen), ‘monogynous’ (one queen) and ‘polygynous’ (two queens). The polygynous treatment simulated the natural queen‐worker interactions of nests with two to four queens per chamber. Inside workers were collected near or on the brood pile. Outside workers were collected in the foraging arena outside the nest. Each sub‐colony consisted of 176 inside and 176 outside workers, evenly split between large and small individuals (1:1 ratio; Figure 1A), along with similar amounts of eggs and larvae. Workers were visually categorised as large or small based on a single observation. We confirmed that large workers had wider heads– a typical proxy for ant body size– than small workers (Welch's *t*‐test: *N* = 389, *t* = 19.037, df = 368.71, *p* < 0.0001; see Supplement for details). Workers were then randomly assigned to each sub‐colony. Each replicate set received a unique ID, each referred to as ‘colony ID’. Since it was not feasible to initiate all replicate sets simultaneously, the experiment was conducted in six different phases, each referred to as a ‘cohort’. The six cohorts started 4 weeks apart with four cohorts containing two replicate sets each and two cohorts with one replicate set each. Ants were maintained in their treatments for 58 days, with worker mortality documented twice per week by counting and removing dead workers. Pupae were removed before emergence. Based on similar studies (Majoe et al. 2021; Choppin, Feldmeyer, and Foitzik 2021; Kohlmeier et al. 2017) and pilot observations of the laboratory set‐up, we chose a 58‐day duration for this experiment. We focused on large workers to compare ovarian development and gene expression between inside and outside workers in response to queen number, avoiding additional complexity resulting from including different worker morphs that did not address our research questions. After 58 days, large inside and outside workers were randomly chosen from each treatment (two samples per worker location, *N* = 12) within one replicate set per cohort (6 replicate sets in total) and frozen at −80°C. Ovary and fat body dissections were performed simultaneously, with dissections and RNA extractions (see Supplement for details) conducted randomly (Figure 1D).

To further explore the role of age in reproduction and gene expression, we conducted a complementary experiment on queens. Queens collected in 2020 were at least 16 months old and likely older, as they were already wingless at collection and were classified as ‘old’ in our transcriptomic analysis. Alate queens collected in 2021 were not older than 3 months when sampled and classified as ‘young.’ After the young queens became reproductive, we set up 12 sub‐colonies, each with one young and one old queen (24 queens total) and 200 large and small workers from inside and outside the nests from both collection years. The queens were marked with thin wires of different colours to denote their age. The queens were maintained in these standardised sub‐colonies for 14 days to ensure a controlled environment for all queens prior to sampling. Thereafter, the queens were frozen at −80°C for later ovary, brain and fat body dissection for fecundity and gene expression analyses.

### 2.3 Oxidative Stress Experiment

After the 58‐day survival experiment, we randomly selected 10 large and 10 small inside workers, as well as 10 large and 10 small outside workers, from each treatment (8 replicate sets; four cohorts). The workers were colour‐marked with paint markers (Edding 750) and kept in small plastic boxes (10 cm × 10 cm × 5 cm). We randomly assigned five large and five small inside workers, five large and five small outside workers to either the control or the oxidative stress treatment (Figure 1C). The next day, workers assigned to the oxidative stress treatment (480 workers per treatment) were subjected to a 0.46 M solution of Paraquat dichloride (CH_3_(C_5_H_4_N)_2_CH_3_・2Cl) dissolved in Millipore water. Paraquat is an herbicide inducing oxidative stress through superoxide formation (Cousin et al. 2013). We applied paraquat solution twice to the dorsal surface of each worker's head using a size 1 Vernissage paintbrush. Head width correlates with body size in ants and determined the approximate amount applied. This method was adapted from Majoe et al. (2021), who successfully used similar concentrations and application protocols across ant species of varying sizes. After application, each ant was isolated for 2 h in a 1.5 mL Eppendorf tube to avoid the transfer of the solution to other ants via trophallaxis (mouth‐to‐mouth fluid exchange), and to facilitate self‐grooming and ingestion, as Paraquat requires ingestion to manifest its effects. Workers of the control treatment were treated similarly but pure Millipore water instead of the paraquat solution was applied. These treatments were repeated every 2 days over a period of 8 days and worker survival was monitored three times per day (Majoe et al. 2021).

### 2.4 Ovary Dissections

To assess the impact of queen number and worker location on ovarian development, we dissected 144 large inside and outside workers under a stereomicroscope (Leica S9i, Microsystems CMS GmbH, Wetzlar, Germany) with magnification ranging from 1.25x to 5.0x. Images (2592 × 1944 px) of the ovaries were captured using Leica LAS software (v.4.12.0). We measured the ovariole length of 129 workers using ImageJ2 Fiji software (v.2.9.0). The mean of the two longest ovarioles was calculated (typically four ovarioles per worker, Figure S1) and the total number of developing oocytes was counted (Fiji software). To assess the impact of age on queen fecundity, we dissected 24 queens and measured mean ovariole length as described for the workers. The ovaries from one young and one old queen were lost during dissections, resulting in 22 measured queen ovaries. We also counted the number of oocytes (Leica LAS software). All queens had sperm‐filled spermathecae.

### 2.5 Statistical Analyses

The *coxme* package (v.2.2–18.1; Therneau 2020) in RStudio (R v.4.2.3; R Core Team 2018) was used to build two Cox‐regression mixed‐effects models. One model was created to analyse the 58‐day survival experiment and one model for the oxidative stress experiment. The variable ‘queen number’ (queenless/ monogynous/ polygynous) was included as fixed factor, and ‘experimental box’ (a combination of ‘colony ID’ and ‘cohort’) as random factor to account for variability associated with both factors. To compare whether worker survival depended on queen number, pairwise comparisons were performed for: queenless versus monogynous; queenless versus polygynous; and monogynous versus polygynous (Holm‐Bonferroni adjusted *p*‐values).

For the oxidative stress experiment, we first constructed a Cox‐regression mixed‐effects model to assess how treatment (oxidative stress/ control) influenced worker survival, accounting for potential interactions with queen number, worker location (inside/ outside) and worker size (large/ small). Fixed factors included ‘treatment’, ‘queen number’, ‘worker location’ and ‘worker size’ and their interactions with treatment. ‘Experimental box’ was included as random factor. To investigate worker survival in each treatment group (oxidative stress/ control) separately, we constructed additional Cox‐regression mixed‐effects models for each treatment. These models included ‘queen number’, ‘worker location’, ‘worker size’ and their interactions as fixed factors, allowing us to assess how worker location and size influenced survival under varying queen numbers in each treatment. ‘Experimental box’ was included as random factor. Non‐significant interactions were removed and the package *emmeans* v.1.10.2 (Lenth 2024) was used to perform pairwise comparisons between groups. Hypothesis testing was performed using the ‘Anova’ function from the package *car* (v.3.1–2; Fox and Weisberg 2019). The package *ggplot2* (v.3.4.2; Wickham 2016) was used to plot the Kaplan–Meier survival curves. All model fits (58‐day survival and oxidative stress experiment) were assessed by visualising the distribution of fitted values using histograms, Quantile–Quantile plots (Q–Q‐plots; R function qqnorm) and overlaid Q‐Q lines (R function qqline) to identify deviations from expected distributions and confirm model appropriateness.

We modelled the mean ovariole length of large inside and outside workers using a linear mixed‐effects model from the package *lme4* (v.1.1–33; Bates et al. 2015) and tested the effect of the explanatory variables with the ‘Anova’ function (*car* package). The number of oocytes in development was modelled using a generalised linear mixed‐effects model (*lme4* package) with a ‘Poisson’ distribution and tested with the ‘Anova’ function. All models included ‘queen number’, ‘worker location’ and their interaction as fixed effects, with ‘sub‐colony ID’ as random factor. Each sub‐colony ID inherited colony ID and cohort information, accounting for variability associated with both factors. The mean ovariole length and the number of oocytes of queens were analysed using a linear mixed‐effects model with the *lme4* package. ‘Mean ovariole length’ and ‘oocyte number’, respectively, were included as the response variable, ‘queen age’ (young/ old) as fixed factor and ‘colony ID’ as a random factor. Hypothesis testing was performed with the ‘Anova’ function. We performed diagnostic checks on the residuals for all models (for workers and queens), including Q–Q plots (R function qqnorm) of the residuals and overlaid Q–Q lines (R function qqline) to ensure model appropriateness. For all analyses, we used a significance threshold of *p* < 0.05.

### 2.6 Differential Expression Analysis

For the differential expression analysis, we used 72 worker samples (fat body) and 23 queen samples (brain and fat body). Detailed information on RNA extraction and sequencing can be found in the Supplement. Raw RNAseq read quality was assessed and filtering was performed to remove adaptors using Fastp (v.0.2; Chen et al. 2018). Post‐filtered sequence quality was assessed using FastQC (v.0.11.8; Andrews 2010) with the data being of high quality to perform differential expression analyses. Since there is no reference genome assembly available for *Tapinoma magnum*, we generated a *de novo* transcriptome assembly using Trinity (v.2.13.2; Grabherr et al. 2011) with the SuperTranscripts option (Davidson et al, 2017). To generate the transcriptome assembly, we used a combination of the filtered queen and worker reads. To quantify transcript abundance, RSEM (v.1.3.3; Li and Dewey 2011) was used, resulting in an overall alignment rate of 79.4% ± 1.5% (mean ± sd) for the worker dataset and 76.7% ± 2.5% (mean ± sd) for the queen dataset. One old queen brain sample was removed from further analyses due to a low mapping rate of 47%, leaving 23 queen samples. Differential gene expression analysis was conducted in RStudio (R v.4.2.3). For each dataset, transcript abundances were introduced into estimated count matrices with the package *tximport* (v.1.26.1; Soneson, Love, and Robinson 2015), serving as input for differential gene expression analyses using the *DESeq2* package (v.1.38.3; Love, Huber, and Anders 2014). Subsequent transcriptome‐based analyses were conducted separately for the worker and queen dataset.

For the worker dataset, contigs with zero reads in at least 11 of 12 replicates were removed from the count matrix, resulting in 73,277 contigs retained in the differential expression analysis. We first examined whether the impact of queen number on gene expression differed between inside and outside workers as we expected inside workers to react more strongly to varying queen numbers than outside workers due to their closer interactions with the queen. To identify genes whose expression was affected by the interaction between queen number and worker location, we compared the following models:

Full model: ~colony ID+ queen number + worker location+ queen number: worker location.

Reduced model: ~colony ID+ queen number + worker location.

Second, to test the main effect of queen number, we compared these models:

Full model: ~colony ID+ worker location+ queen number.

Reduced model: ~colony ID+ worker location.

Third, to examine the main effect of worker location on gene expression, we compared the following models:

Full model: ~colony ID+ queen number + worker location.

Reduced model: ~colony ID+ queen number.

To identify groups of differentially expressed genes with similar expression patterns, we conducted a cluster‐based analysis with the R package *DEGreport* (v.1.34.0; Pantano 2017).

For the queen‐based analysis, separate analyses were performed for each tissue. Only contigs with more than zero reads in at least five out of six replicates per tissue were kept, resulting in 55,349 contigs for the differential expression analysis. The following model comparison was performed to address our research question:

Full model: ~age.

Reduced model: ~1.

All model comparisons were conducted using Likelihood Ratio Tests (LRT) as implemented in *DESeq2* with Benjamini‐Hochberg adjusted *p*‐values of 0.05 used as a threshold to determine whether a gene was significantly differentially expressed.

To explore the relationship between age and gene expression in workers and queens, and to assess if worker location can serve as a proxy for age, we compared differentially expressed genes that were upregulated in inside or outside workers with those upregulated in young or old queens using Venny (v.2.1; Oliveros 2007–2015). Fisher's exact tests (significance threshold *p* < 0.05) examined the significance of overlaps between inside workers versus young queens, outside workers versus old queens, inside workers versus old queens and outside workers versus young queens, using a total pool of 52,093 contigs (common number of contigs in workers and queens).

### 2.7 Gene Annotation and Functional Enrichment Analyses

To annotate the Trinity‐assembled transcriptome, we conducted a BlastX homology search against the non‐redundant invertebrate protein database (Altschul et al. 1990; downloaded July 2023). Nucleotide sequences were translated into predicted amino‐acid sequences using TransDecoder (v.5.7.0‐Perl‐5.30.0; Haas 2017), which were then used as input for InterProScan (v.5.54–87.0; Blum et al. 2021) for the identification of conserved functional domains and assignment of associated Gene Ontology (GO) terms to our *de novo* transcriptome assembly. We additionally used OrthoFinder (v.2.5.4; Emms and Kelly 2019) to examine potential homologues between the closely related dolichoderine ant *Linepithema humile* (Ensembl Metazoa Biomart) and *Tapinoma magnum*, resulting in the identification of 5859 potential homologues, of which 63% could be assigned additional GO terms based on homology to *L. humile* . We combined both sets of GO terms to create a GO term database, which was then used in our GO term enrichment analysis. To identify which terms attributed molecular functions functionally enriched within workers and queens, we performed individual GO term enrichment analyses based on the differentially expressed contigs using Fisher's exact tests (significance threshold *p* < 0.05) implemented by the package *TopGo* (v.2.50.0; Alexa and Rahnenführer, 2010) using the weight01 algorithm. The scripts used for these analyses were modified versions of those developed by Colgan et al. (2019).

## 3 Results

### 3.1 Queen Number Influenced Worker Survival, While Worker Location Was Associated With Ovarian Development

**FIGURE 2:**
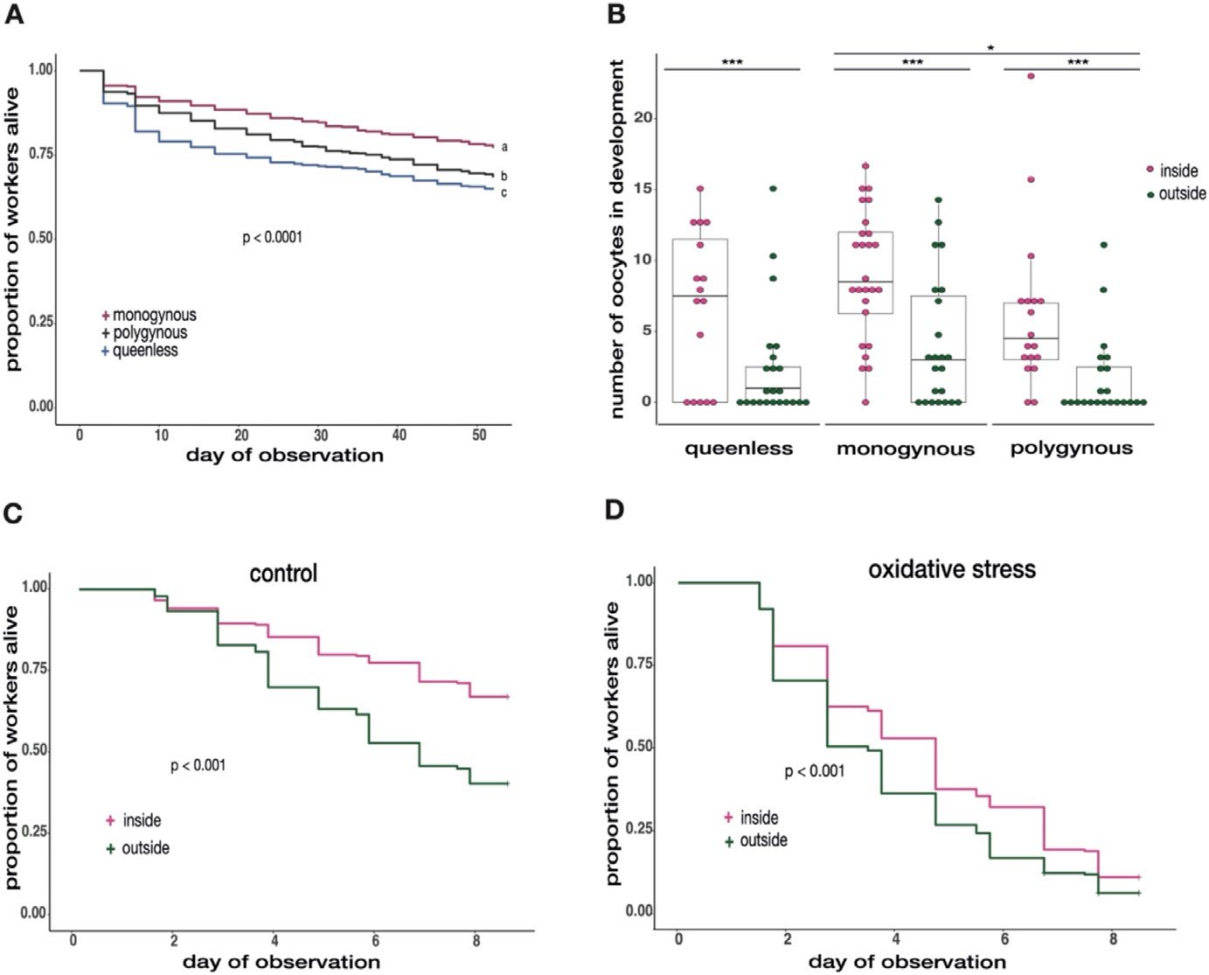
Worker survival, ovarian development and oxidative stress resistance dependent to queen number and worker location. (A) Worker survival over 58 days under queenless, monogynous and polygynous conditions. Workers of monogynous (red line) colonies survived best, followed by workers of polygynous (grey line) colonies and workers of queenless (blue line) colonies (Queen number 0 vs. 1: *χ* ^2^ = 155, *p* < 0.001; 0 vs. 2: *χ* ^2^ = 41.6, *p* < 0.001; 1 vs. 2: *χ* ^2^ = −51.7, *p* < 0.001).(B) The number of oocytes dependent on queen number and worker location. Inside (pink) workers had more oocytes in development than outside (green) workers (GLMER: *χ* ^2^ = 77.11, *p* < 0.0001). Workers of monogynous colonies developed more oocytes than workers of polygynous colonies (GLMER: *χ* ^2^ = 4.32, *p* = 0.037), with an interaction between queen number and worker location (GLMER: *χ* ^2^ = 6.65, *p* = 0.009). Asterisks indicate levels of significance: *p* < 0.05 (*), *p* < 0.01 (**), *p* < 0.001 (***). (C) Worker survival in the control treatment (oxidative stress experiment). Inside (pink line) workers had a higher survival probability than outside (green line) workers (*χ* ^2^ = 37.78, *p* < 0.0001). (D) Worker survival in the oxidative stress treatment. Inside workers showed a higher resistance to oxidative stress than outside workers (*χ* ^2^ = 26.81, *p* < 0.0001).

To investigate the effect of queen number on worker survival, we monitored worker survival in queenless, monogynous and polygynous colonies over 58 days. We found that queen number influenced worker survival, as workers from monogynous sub‐colonies survived best, followed by workers of polygynous colonies, and then workers of queenless colonies (queen number 0 vs. 1: χ^2^ = 155, *p* < 0.001; 0 vs. 2: *χ* ^2^ = 41.6, *p* < 0.001; 1 vs. 2: *χ* ^2^ = 51.7, *p* < 0.001; Figure 2A). We further examined the influence of queen number and worker location on ovarian development by measuring ovariole length and counting the number of developing oocytes in large inside and outside workers from each queen treatment. The workers had a mean ovariole length of 1.86 mm (± 0.4 mm sd), which was independent of queen number and worker location (LMER_queen number_: χ^2^ = 1.17, *p* = 0.56; LMER_worker location_: *χ* ^2^ = 2.75, *p* = 0.10; Figure S2). In contrast, inside workers had more developing oocytes than outside workers (GLMER: *χ* ^2^ = 77.11, *p* < 0.0001; Figure 2B). Moreover, workers of monogynous sub‐colonies had more oocytes than workers of polygynous sub‐colonies (GLMER: *χ* ^2^ = 4.32, *p* = 0.037; Figure 2B), with an interaction between queen number and worker location (GLMER: *χ* ^2^ = 6.65, *p* = 0.009).

### 3.2 Worker Location but Not Queen Number Influenced Worker Resistance to Oxidative Stress

To test whether queen number, worker location and worker size influenced the resistance of workers to oxidative stress, we subjected large and small inside and outside workers from each queen treatment to either a paraquat‐induced oxidative stress treatment or the control treatment (Millipore water). The oxidative stress treatment reduced worker survival compared to the control treatment (*χ* ^2^ = 306.96, *p* < 0.0001), while queen number did not influence worker survival in both treatment groups (*χ* ^2^ = 1.53, *p* = 0.466). We found significant interactions between the treatment groups and the fixed factors queen number (*χ* ^2^ = 6.986, *p* = 0.03), worker location (*χ* ^2^ = 8.769, *p* = 0.003) and worker size (*χ* ^2^ = 6.261, *p* = 0.012) and subsequently investigated worker survival within each treatment group separately. In the control treatment, we found weak evidence that queen number influences worker survival (*χ* ^2^ = 4.79, *p* = 0.091). Closer examination revealed that this trend followed a similar pattern as to what we observed after our 58‐day survival experiment (queen number 0 vs. 1: *z* = 1.808, *p* = 0.167; 0 vs. 2: *z* = −0.242, *p* = 0.968; 1 vs. 2: *z* = −2.043, *p* = 0.102). Inside workers survived better than outside workers in both, the control and the oxidative stress treatment (control: *χ* ^2^ = 37.78, *p* < 0.0001; oxidative stress: *χ* ^2^ = 26.81, *p* < 0.0001, Figure 2C/D), while worker survival in the control was lower compared to the 58‐day survival experiment (Figure 2A). Additionally, large workers survived better under oxidative stress than small workers (*χ* ^2^ = 46.11, *p* < 0.001), while we found very weak evidence that worker size influenced survival in the control treatment (*χ* ^2^ = 2.69, *p* = 0.10; Figure S3).

### 3.3 Worker Location Rather Than Queen Number Influenced Fat Body Gene Expression in Workers

**FIGURE 3:**
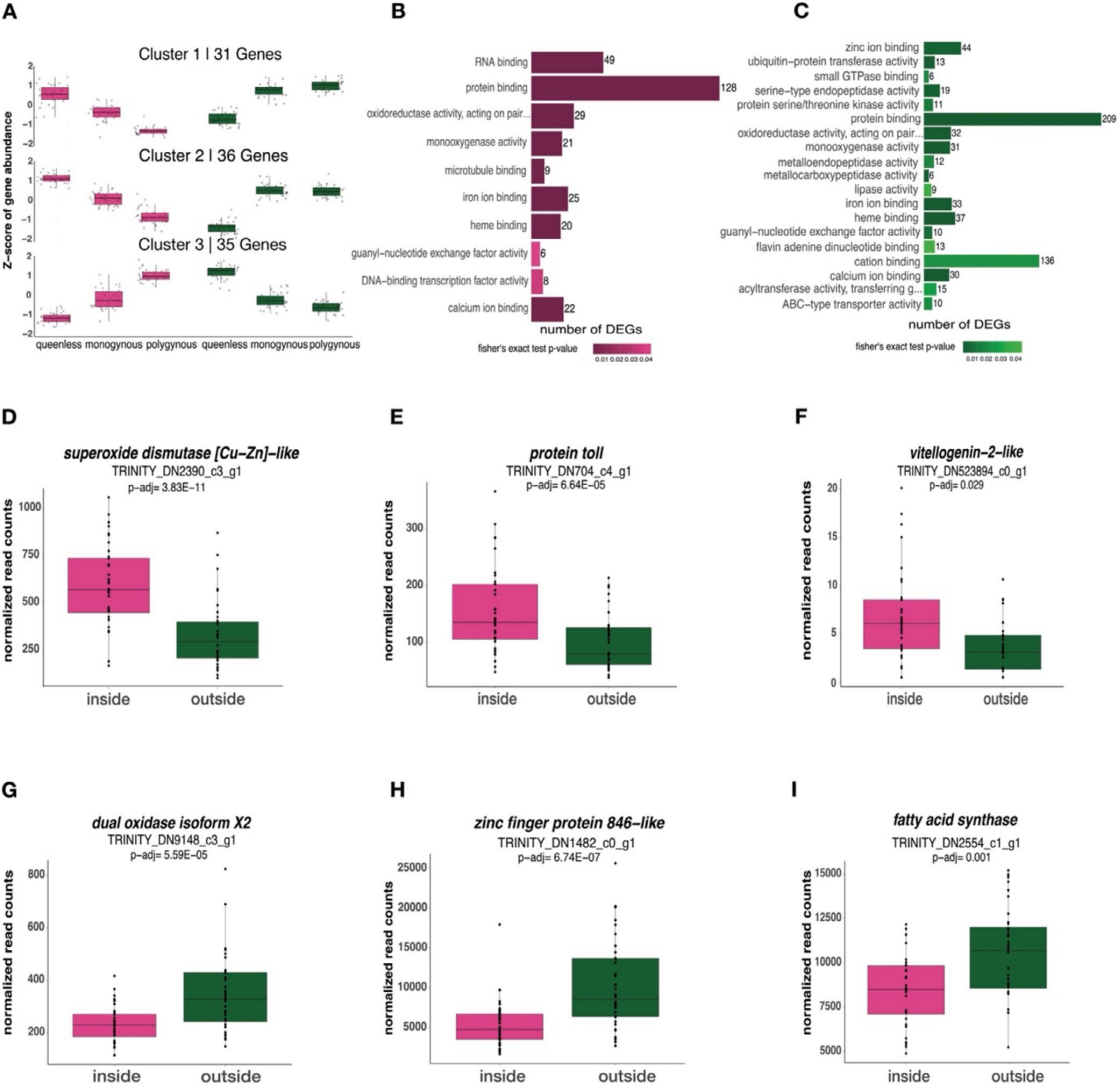
The influence of queen number and worker location on fat body gene expression. (A) 102 out of the 108 DEGs from the interaction term (queen number: Worker location) were grouped into three clusters based on the expression profiles within each worker caste (pink = inside workers; green = outside workers) and social environment. (B) The 10 most enriched GO terms (molecular function) in the list of genes upregulated in inside workers and (C) the 19 most enriched GO terms (molecular function) in the list of genes upregulated in outside workers. The size of each bar correlates with the number of DEGs associated with each term, while the colour correlates with the *p*‐value (the darker the colour, the lower the *p*‐value). (D–I) Selected DEGs with notable function within inside workers and outside workers.

To investigate the effect of queen number and worker location on fat body gene expression (72 samples), we analysed gene expression differences between inside and outside workers across treatments (queenless, monogynous, polygynous). We found no evidence that queen number affected fat body gene expression (0 differentially expressed genes, all BH‐adjusted *p*‐value > 1), while worker location was linked to strong transcriptomic differences. Between inside and outside workers, we identified 4305 significantly differentially expressed genes (DEGs; BH‐adjusted *p* < 0.05). Of these, 2188 showed an elevated expression in inside workers, while 2117 DEGs were elevated in outside workers. We identified 108 DEGs, whose expression was affected by an interaction between worker location (inside/ outside) and queen number (queenless/ monogynous/ polygynous), of which, 102 were grouped into three co‐expression clusters (Figure 3A). The first cluster consisted of 31 genes whose expression in inside workers decreased with increasing queen number. Within outside workers the expression was lower in queenless colonies and was similarly increased in monogynous and polygynous colonies. Cluster 2 consisted of 36 genes where expression was highest in inside workers sampled from queenless colonies, while, similar to cluster 1, the expression was decreasing with queen number. In outside workers, the difference in expression between queenless and monogynous colonies was even stronger compared to cluster 1. The expression in monogynous and polygynous colonies was similarly high. The blast homology search identified two genes in cluster 1 (*serine protease inhibitor ¾‐like isoform X3*, LRT: BH‐adjusted *p* = 0.048; *group XIIA secretory phospholipase A2*, LRT: BH‐adjusted *p* = 0.049) and two genes in cluster 2 (*ribosome biogenesis protein WDR12 homologue*, LRT: BH‐adjusted *p* = 0.049; *cell division cycle protein 123 homologue isoform X1*, LRT: BH‐adjusted *p* = 0.037). Finally, cluster 3 contained 35 genes with expression patterns opposite to the first two clusters (Figure 3A). In inside workers, expression profiles increased with queen number, while outside workers showed the highest expression in queenless colonies, which was decreasing in monogynous and polygynous colonies. Here, we found nine genes related to immunity and detoxification (e.g., *xanthine dehydrogenase 1‐like*, LRT: BH‐adjusted *p* = 0.037; *glucose dehydrogenase*, LRT: BH‐adjusted *p* = 0.047; Kim et al. 2001; Lee et al 2005), three genes involved in the Ras signalling pathway (e.g., *ras‐like GTP‐binding protein Rho1 isoform X2*, LRT: BH‐adjusted *p* = 0.013), and *juvenile hormone epoxide hydrolase 1‐like* (LRT: BH‐adjusted *p* = 0.047).

Genes with higher expression in inside workers were enriched for 10 Gene Ontology (GO) terms (Figure 3B, Table S1), with the most significantly enriched terms being *oxidoreductase activity* (Fisher's exact *p* < 0.0001), *iron ion binding* (Fisher's exact *p* < 0.0001) and *monooxygenase activity* (Fisher's exact *p* < 0.0001). In contrast, 19 enriched GO terms were identified for DEGs upregulated in outside workers. Next to terms also represented in inside workers, we identified terms related to lipid metabolic processes (e.g., *lipase activity*, Fisher's exact *p* = 0.036) and proteolysis (e.g., *metallocarboxypeptidase activity*, Fisher's exact *p* < 0.001), drawing a more diverse expression profile in outside workers (Figure 3C; Table S2).

**FIGURE 4:**
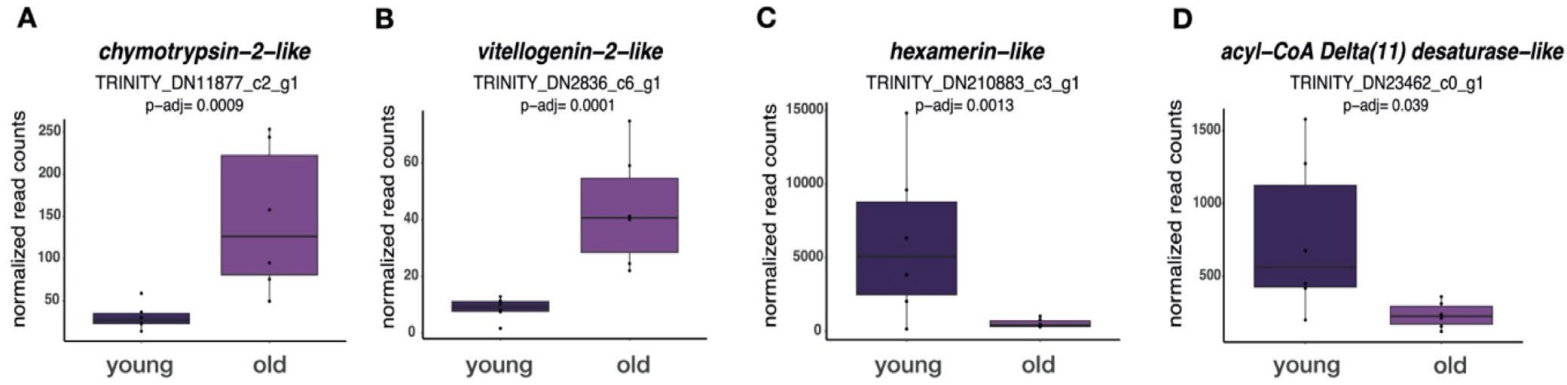
Candidate DEGs of interest differing in expression in the fat body between young and old queens. (A) Normalised read counts of *chymotrypsin‐2‐like* and (B) *vitellogenin‐2‐like* which are significantly overexpressed in old queens. (C) Normalised read counts of *hexamerin‐like* and (D) *acyl‐Coa Delta*(*11*) *desaturase‐like* which are overexpressed in young queens.

Within the genes upregulated in inside workers, our blast homology search identified the antioxidants *superoxide dismutase [Cu‐Zn]‐like* (LRT: BH‐adjusted *p* = 3.83E‐11, Figure 3D), *transferrin* (LRT: BH‐adjusted *p* = 0.003), as well as *protein toll* (LRT: BH‐adjusted *p* = 6.64E‐05, Figure 3E). Moreover, we found *vitellogenin‐1* (LRT: BH‐adjusted *p* = 0.045), *vitellogenin‐1‐like* (LRT: BH‐adjusted *p* = 0.012) and *vitellogenin‐2‐like* (LRT: BH‐adjusted *p* = 0.029, Figure 3F; all *conventional vitellogenins*, *C‐Vg*; Kohlmeier, Feldmeyer, and Foitzik 2018). Within the genes upregulated in outside workers, we found higher expression of *dual oxidase isoform X2* (LRT: BH‐adjusted *p* = 0.035, Figure 3G), 22 zinc finger proteins (e.g., *zinc finger protein 846‐like*, LRT: BH‐ adjusted *p* = 6.74E‐07, Figure 4H) as well as 24 DEGs involved in lipid metabolism (e.g., *fatty acid synthase*, LRT: BH‐adjusted *p* = 0.001, Figure 3I).

### 3.4 Queen Age Elicited no Changes in Fecundity but Minor Transcriptional Shifts in Fat Body Gene Expression

To investigate the effect of age on queen fecundity and gene expression, we dissected the ovaries (22 queen ovaries) and analysed brain and fat body gene expression of young and old *T. magnum* queens (23 samples in total). Age did not influence fecundity as young queens had similarly long ovarioles and as many oocytes in development as old queens (LMER_ovariole length_: χ^2^ = 0.02, *p* = 0.811; LMER_oocyte number_: χ^2^ = 0.04, *p* = 0.836; Figure S4).

No age‐related transcriptomic changes were detected in the brain (11 samples). However, 126 DEGs were identified in the fat bodies between young and old queens (12 samples). Of these, 75 DEGs were upregulated in old queens, while 51 DEGs were upregulated in young queens. In old queens, we found an overexpression of *chymotrypsin‐2‐like* (LRT: BH‐adjusted *p* = 0.0009, Figure 4A), *vitellogenin‐1‐like* (LRT: BH‐adjusted *p* = 0.005) and *vitellogenin‐2‐like* (LRT: BH‐adjusted *p* = 0.0001; Figure 4B; *C‐Vg*; Kohlmeier, Feldmeyer, and Foitzik 2018). Young queens showed increased expression of *hexamerin‐like* (LRT: BH‐adjusted *p* = 0.0013, Figure 4C) and *acyl‐CoA Delta*(*11*) *desaturase‐like* (LRT: BH‐adjusted *p* = 0.039, Figure 4D). We could not identify significantly enriched GO terms within the DEGs between young and old queens.

To explore the link between age and gene expression, we compared DEGs that were upregulated in young and old queens with those upregulated in inside and outside workers. We found that 20 DEGs were upregulated in inside workers and young queens and 15 DEGs were upregulated in outside workers and old queens. These overlaps were larger than what could be expected by chance (Fisher's exact test *p* < 0.0001; Fisher's exact test *p* < 0.0001, respectively). Hexamerin storage proteins were enriched in inside workers and young queens. We found four genes to be upregulated in both inside workers and old queens and five in outside workers and young queens and we cannot reject the hypothesis that these overlaps may be explained by chance (Fisher's exact test *p* = 0.558, Fisher's exact test *p* = 0.055, respectively; see Supplement for details).

## 4 Discussion

### 4.1 Physiological and Transcriptional Effects of Queen Number and Worker Location

We investigated whether queen number and worker location affected worker survival, ovarian development, oxidative stress resistance, as well as fat body gene expression in the invasive ant *Tapinoma magnum*. Our two‐month survival experiment revealed that workers of monogynous colonies survived best, followed by workers of polygynous colonies, with the lowest survival in workers of queenless colonies. Workers of monogynous colonies had more oocytes in development compared to those from polygynous colonies, while inside workers had more oocytes than outside workers. Oxidative stress resistance and fat body gene expression were not directly influenced by queen number whereas worker location was strongly linked to both.

Our results differ from previous studies on other ant species, such as *Temnothorax longispinosus* (Kohlmeier et al. 2017), *Acromyrmex echinatior*, *Atta colombica* and *Temnothorax rugatulus* (Majoe et al. 2021). Specifically, *T. magnum* workers exhibited the highest mortality in queenless colonies, contrasting with the increased lifespan observed in queenless workers of these species. While worker reproduction has been observed in the invasive ant *Anoplolepis gracilipes* (Lee et al. 2017), it remains unclear whether worker reproduction increases following queen loss or how it impacts worker lifespan. More recently, Hamidi, de Biseau, and Quinet (2023) demonstrated that workers of the highly polygynous ant *Crematogaster pygmaea* can produce males in the absence of queens. However, the potential link between this reproductive investment and worker lifespan remains uncertain. The effects of queen loss in strictly polygynous species with functionally sterile workers remain understudied. Our results indicate that queen loss decreases worker survival, without influencing ovarian development. While the complete loss of all queens in a supercolony is highly unlikely, queenlessness in a single nest chamber is more probable and can cause stress to the workers. Consequently, increased metabolic rates and ROS production may increase mortality (Pearl 1928). Although *T. magnum* is highly polygynous, our results demonstrate that workers respond to differences in queen number, indicating that workers are sensitive not only to queen presence but also to the number of queens in their local nest.

The higher survival in monogynous colonies might be linked to the increased oocyte development and presumably increased trophic egg‐laying in workers kept in monogynous compared to polygynous conditions. Trophic eggs provide essential nutrients, especially proteins, for growth and reproduction in larvae and queens (Gobin, Peeters, and Billen 1998; Gobin and Ito 2000). In monogynous colonies, egg production hinges on the sole queen. Queen fecundity and nutritional care provided to the queens are likely correlated (Hannonen et al. 2002; Trettin et al. 2011; Chen and Vinson 2000), while queens may receive less food under polygynous conditions compared to monogynous conditions (Keller 1988). Increased trophic egg‐laying might be linked to a higher nutritional care provided to the single queen. Workers might have increased their own lifespan to increase the fitness and survival of the single queen, ultimately ensuring colony survival. Alternatively, workers may increase their lifespans by shifting to intranidal tasks to support the colony's needs (Schultner, Oettler, and Helantera 2017; Calabi and Traniello 1989; Robinson, Feinerman, and Franks 2009).

Queen number did not directly affect worker survival under oxidative stress, though very weak evidence suggested workers from monogynous conditions survived better in the control treatment. Worker survival in the control treatment was lower than in the 58‐day experiment, likely because the oxidative stress experiment occurred after the two‐month experiment, when workers had a shorter residual lifespan. Differences in oxidative stress resistance were primarily linked to worker location, supporting our hypothesis that younger inside workers invest more into body repair. Since outside workers are likely older, this investment may not be worthwhile given their shorter residual lifespan (Kohlmeier et al. 2017; Majoe et al. 2021). Alternatively, the pronounced impact of paraquat exposure may have masked any potential effects associated with queen number.

Our transcriptomic analysis revealed that inside and outside workers responded with divergent transcriptional shifts to queen number. Genes of interest in the first cluster were *serine protease inhibitor ¾‐like isoform X3* and *group XIIA secretory phospholipase A2*. Serine protease inhibitors are involved in immune responses (Kanost 1999; Kanost and Clarke 2005), while the inhibition of phospholipases A2 (PLA_2_) impairs egg‐laying behaviour, metabolism and immunity in insects (Stanley and Kim 2019). In queenless colonies, these genes were among those expressed the most by inside workers and less by outside workers. In cluster 2, genes of interest were related to detoxification (*ribosome biogenesis protein WDR12 homologue*) and the cell division cycle (*cell division cycle protein 123 homologue isoform X1*). Cell cycle division proteins are involved in various biological processes and are often increased after injuries (Pal and Raj 2022). Thus, inside workers may exhibit a more pronounced response to queenlessness, potentially through the activation of stress‐ and immunity‐related genes. Older outside workers likely faced increased mortality regardless of queen number. This supports our hypothesis that worker responses to queen number vary depending on their location within the nest and possibly their age. In cluster 3, we found more genes related to immunity and detoxification as well as Ras proteins (e.g., *ras‐like GTP‐binding protein Rho1 isoform X2*) and *Juvenile hormone epoxide hydrolase 1‐like* (*JHEH*), upregulated in inside workers within monogynous and polygynous colonies compared to queenless colonies, while outside workers overexpressed these genes under queenless conditions. Ras proteins play a central role in cell cycle regulation and tissue repair (Boonstra et al. 1995; Stacey 2003). *JHEH* plays an important role in the degradation pathways of juvenile hormone (JH). In adult females of *Drosophila melanogaster*, JH is required for oogenesis and reproductive maturation (Dubrovsky, Dubrovskaya, and Berger 2002). Moreover, gene expression varied less in outside workers within monogynous and polygynous colonies in all three clusters.

Worker location was associated with strong changes in gene expression, likely further explaining the higher resistance to oxidative stress in inside workers. Inside workers invested more into antioxidant genes (*superoxide dismutase [Cu‐Zn]‐like* and *transferrin*), accompanied by an overexpression of *vitellogenin‐2‐like* (*C‐*Vg; Kohlmeier, Feldmeyer, and Foitzik 2018) and *protein toll‐like*. Superoxide dismutase and transferrin are known to eliminate ROS while they are also linked to the individual's reproductive potential (Nojima et al. 2015; Tasaki et al. 2018). Vg proteins are synthetized in the fat body and secreted into the haemolymph before they are transported into the oocytes (Hagedorn and Kunkel 1979). Elevated *Vg* expression is typically found in queens but can also be found in younger inside workers compared to older outside workers (Corona et al. 2007; Wu et al. 2021), potentially linked to the increased oocyte development in inside workers. Moreover, the insect Toll pathway and its receptors play an important role in both, immunity and development (Evans et al. 2006).

In outside workers, we found the overexpression of *dual oxidase isoform X2*, *zinc finger proteins* and *fatty acid synthase*. Dual oxidase induces oxidative stress to challenge gut microbiome bacteria (Ha et al. 2005; Sistermans et al. 2023), while zinc is involved in regulatory pathways (Kandel 2009; Klug 2005). Fatty acid synthase, involved in energy metabolism and promoting survival in older animals (Chaudhari and Kipreos 2018), may help compensate cellular damage from ageing in outside workers, supporting the hypothesis that they were older, consistent with age‐related division of labour in social insects.

While we acknowledge that precise control of worker age was not possible in our study, our analysis revealed significant overlaps in upregulated genes between inside workers and young queens and between outside workers and old queens. This suggests a link between worker location and age. The upregulation of hexamerin storage proteins in inside workers and young queens not only indicates that inside workers were indeed younger than outside workers but also suggests that both groups store proteins to produce reproductive or trophic eggs. Additionally, the greater oxidative stress resistance, the investment in antioxidants, and increased egg‐laying potential in inside workers suggest a link between worker age, egg production and task. In *Temnothorax* ants, worker task is more strongly linked to gene expression than age and ovarian development (Kohlmeier et al. 2019). Although we used age polyethism (Wilson 1971) to infer worker age based on location it is also possible that reproductive status influences their location, as workers with higher reproductive potential (i.e., an individual's ability to develop their ovaries) tend to remain inside the nest (Bourke 1988).

### 4.2 The Physiological and Molecular Influence of Ageing in Queens

We investigated the effects of ageing on fecundity and tissue‐specific gene expression in young and old *T. magnum* queens. We found no evidence that age affected fecundity or brain gene expression, but fat body gene expression varied with age (126 DEGs).

We found a higher expression of *chymotrypsin‐2‐like, vitellogenin‐1‐like* and *vitellogenin‐2‐like* (C‐Vgs; Kohlmeier, Feldmeyer, and Foitzik 2018) in old queens. Chymotrypsin is often expressed as immune response against (bacterial) infection (Viljakainen et al. 2018), possibly linked to an increase in body repair within old queens. In *P. barbatus, vitellogenin 1* expression is higher in queens and nurses, while *vitellogenin 2* is higher expressed in foragers (Corona et al. 2013). These copies might stand in contrast to each other, one indicating a high reproductive potential and the other indicating reproductive senescence in old queens of *T. magnum* . Moreover, the elevated *vitellogenin 2* expressions in both old queens and young workers may reflect a secondary function, such as protection against oxidative stress (Seehuus et al. 2006).

Young queens (and inside workers) overexpressed *hexamerin‐like* and *acyl‐CoA Delta(11) desaturase‐like*. In *Camponotus festinatus* founding queens, high levels of *hexamerin* (storage proteins; Beintema et al. 1994) are related to the production of the first batch of eggs (Martinez and Wheeler 1994). Since nest spots of *T. magnum* may contain over 350 queens (Seifert et al. 2017), the workers might not provide each queen with the same amount of food and care (Hannonen et al. 2002; Keller 1988; Chen and Vinson 2000). Thus, young queens possibly use up their storage proteins to start reproduction. Acyl‐CoA desaturases play a crucial role in synthesising cuticular hydrocarbons (CHCs) by introducing double bonds into fatty acyl‐CoA precursors, leading to the formation of unsaturated hydrocarbons (Hazel and Williams 1990; Miyazaki and Ntambi 2003). In ants, these enzymes are integral to producing CHCs, which serve as chemical signals and protective barriers against desiccation. Notably, the genomes of *Linepithema humile* and *Solenopsis invicta* harbour multiple acyl‐CoA desaturase genes. This gene expansion may enable these species to diversify their chemical profiles, potentially facilitating adaptation to new ecological niches and contributing to their invasive success (Helmkampf, Cash, and Gadau 2015).

Our results contrast with a study on *Temnothorax* ants showing that older queens have better developed ovaries (Negroni, Foitzik, and Feldmeyer 2019). Indeed, differences in queen ovarian development might depend on the age gap between young and old queens (Seistrup et al. 2023). In our study, old queens were at least 1 year older than young queens. Queen lifespan varies among species and correlates with social structure (Keller and Genoud 1997; Kramer et al. 2022). In queens of similarly invasive, polygynous species, queen lifespans may range from 1 to 3 years (Goodisman and Ross 1999; Reuter et al. 2001). Thus, *T. magnum* queens have likely shorter lifespans, potentially explaining the similar ovarian development with age. Similarly, the lack of age‐related fecundity changes may be characteristic of invasive species (Majoe et al. 2024). Contrarily to previous studies (Negroni, Foitzik, and Feldmeyer 2019; Von Wyschetzki et al. 2015), we did not find many age‐related differences in fat body gene expression (~1500 DEGs vs. 126 DEGs). In the supercolonial ant *Lasius neglectus*, similarly subtle age‐dependent shifts in queen gene expression were observed (Majoe et al. 2024; 165 DEGs). The limited variation in age‐related gene expression may correlate with the species' polygynous nature and a shorter queen life expectancy, suggesting stable fecundity and gene expression throughout life (Jaimes‐Nino, Heinze, and Oettler 2022). However, the lack of precise information on queen life expectancy and the varying durations queens of different ages spent under laboratory conditions could have influenced our findings. While queen physiology may deteriorate more rapidly towards the end of their lives, our queens were likely middle‐aged, which may explain the absence of pronounced age‐related changes in gene expression and fecundity (Jaimes‐Nino, Heinze, and Oettler 2022).

## 5 Conclusion

Our study offers novel insights into the physiological and molecular responses of the supercolonial ant *Tapinoma magnum* across varying social structures, revealing distinct life‐history traits compared to non‐invasive species (Kohlmeier et al. 2017; Majoe et al. 2021; Negroni et al. 2021). We found that workers survived better in monogynous colonies than in polygynous ones, while complete queen removal increased mortality, highlighting the complex interplay between social factors and physiology. Physiological and transcriptional changes in workers may be related to their location and align with patterns seen in other non‐invasive ant species (Majoe et al. 2021; Negroni et al. 2021). The increased oocyte development in inside workers and the shared gene expression profiles with young queens suggests that both, egg‐laying potential and age, drive the observed physiological changes, such as enhanced oxidative stress resistance and altered gene expression, though disentangling these factors is challenging. Future research should control for worker age to clarify how location, age and egg‐laying potential affect transcription and stress susceptibility. Contrarily, queen age minimally impacted fecundity and gene expression, suggesting that reproductive activity is maintained with age (Jaimes‐Nino, Heinze, and Oettler 2022).

## Author Contributions

S.F., R.L., M.M. and A.L. designed the experiment. Ants have been collected by S.F., M.M., S.S. and A.L. S.S., supervised by S.F. and co‐supervised by A.L., conducted the survival and the oxidative stress experiment. A.L. analysed the data collected by S.S., dissected and measured ovaries from workers as well as dissected fat bodies and extracted the RNA. M.M. and A.L. dissected brain and fat body from the queens and M.M. helped A.L. with the RNA extractions. S.F., R.L., T.J.C. and M.M. helped A.L. in the statistical analyses and interpretation. T.J.C. and A.L. annotated the transcriptome assembly. A.L. wrote a first draft and all authors commented on it. A.L., S.F., R.L., T.J.C. finalised the manuscript based on these comments. S.F. supervised the experimental parts of the project.

## Conflicts of Interest

The authors declare no conflicts of interest.

## Supporting information


Data S1.

## Data Availability

The raw data and code for the statistical analyses, including annotation files are available through Dryad (https://doi.org/10.5061/dryad.j0zpc86p3). Additional supplemental information (methods, figures, tables) can be found in the supplement. Raw read sequences are accessible through the NCBI BioProject ID PRJNA1120839.
