# Supplementary material for: Worker Survival and Egg Production—But Not Transcriptional Activity—Respond to Queen Number in the Highly Polygynous, Invasive Ant Tapinoma magnum
Source: Mol Ecol. 2025 Feb 4;34(6):e17679. doi: 10.1111/mec.17679 (PMC11874646; doi:10.1111/mec.17679)
Supplement: Supplementary file 1 — Data S1. [file MEC-34-e17679-s001.docx]

**Supplement**

***RNA extraction and Sequencing***

Large inside and outside workers from each experimental box were sampled after the 58-day survival experiments. The workers were frozen at -80°C between 12:00 and 16:00 and their fat bodies were dissected. We took the fat body from four individual workers and pooled their fat bodies to create 72 individual RNA pools. We created two RNA pools per worker location (inside, outside) across the six cohorts, resulting in a total of 12 replicates per worker location. Each pool of four fat bodies was stored in 100µl of TRIzol™ LS Reagent (Invitrogen™) at -80°C until extraction. RNA was extracted in a 4:1 ratio of TRIzol: Chloroform: Isoamylalcohol followed by purification steps using the Qiagen RNA-easy Mini Kit. At the Beijing Genomics Institute (BGI), the High Sensitivity RNA Analysis Kit (Fragment Analyser) was used to check the quality of the extracted RNA. The libraries were prepared and sequenced by BGI using the Illumina HiseqXTen sequencing platform, yielding 150 bp paired- end reads with a sequencing depth of 23.36 ± 3 (mean ± sd) million reads.

For the queen dataset, we sampled and dissected six young and six old queens (12 total queens in total) and transferred brain and abdominal fat body tissues into 100µl of TRIzol™ LS Reagent (N= 12 brain samples; N=12 fat body samples). RNA extractions were conducted as described above and extracted RNA was checked using the Agilent 2100 Bioanalyzer, RNA 6000 Nano Kit at BGI. Library preparation and sequencing were similarly performed by BGI using the Illumina HiseqXTen sequencing platform, yielding in 150 bp paired- end reads with a sequencing depth of 23.58 ± 2.54 (mean ± sd) million reads.

***Head width measurements***

To validate our visual size classification of large and small workers, we collected additional 389 workers, categorized as large or small, from inside and outside the source colony. We measured head widths (the distance between the eyes) using Leica LAS software to determine if large workers were significantly larger than small workers. Consistent with the method used in the experiment set-up, workers were classified based on a single visual observation. A Welch’s t-test was then performed to assess whether the head widths of large and small workers differed significantly.


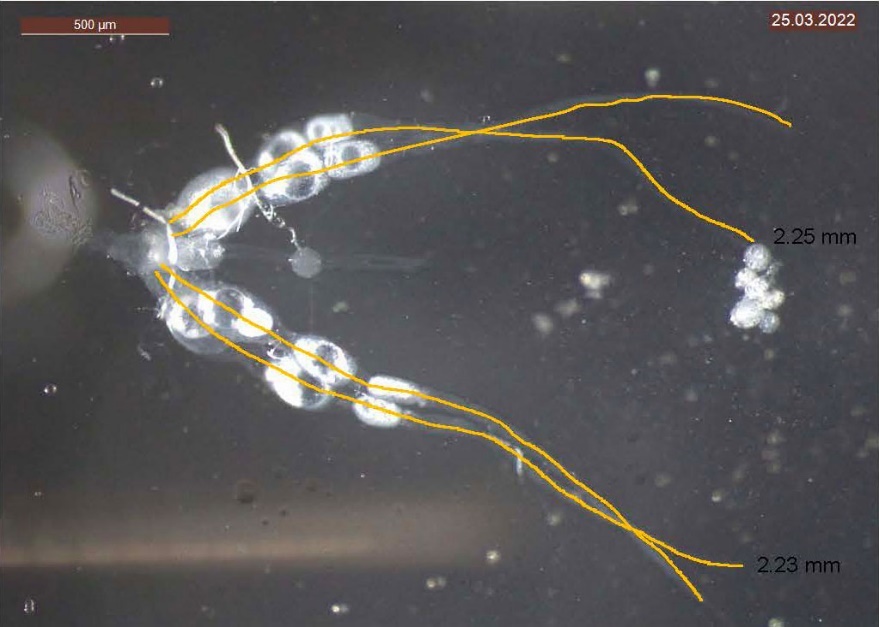


Figure S 1: Image of a worker ovary showing four ovarioles. The orange lines illustrate the measurement paths for the ovarioles. The lengths of the two longest ovarioles were used to calculate the mean ovariole length. The scale bar is shown in the top left corner, and the dissection date is displayed in the top right corner.


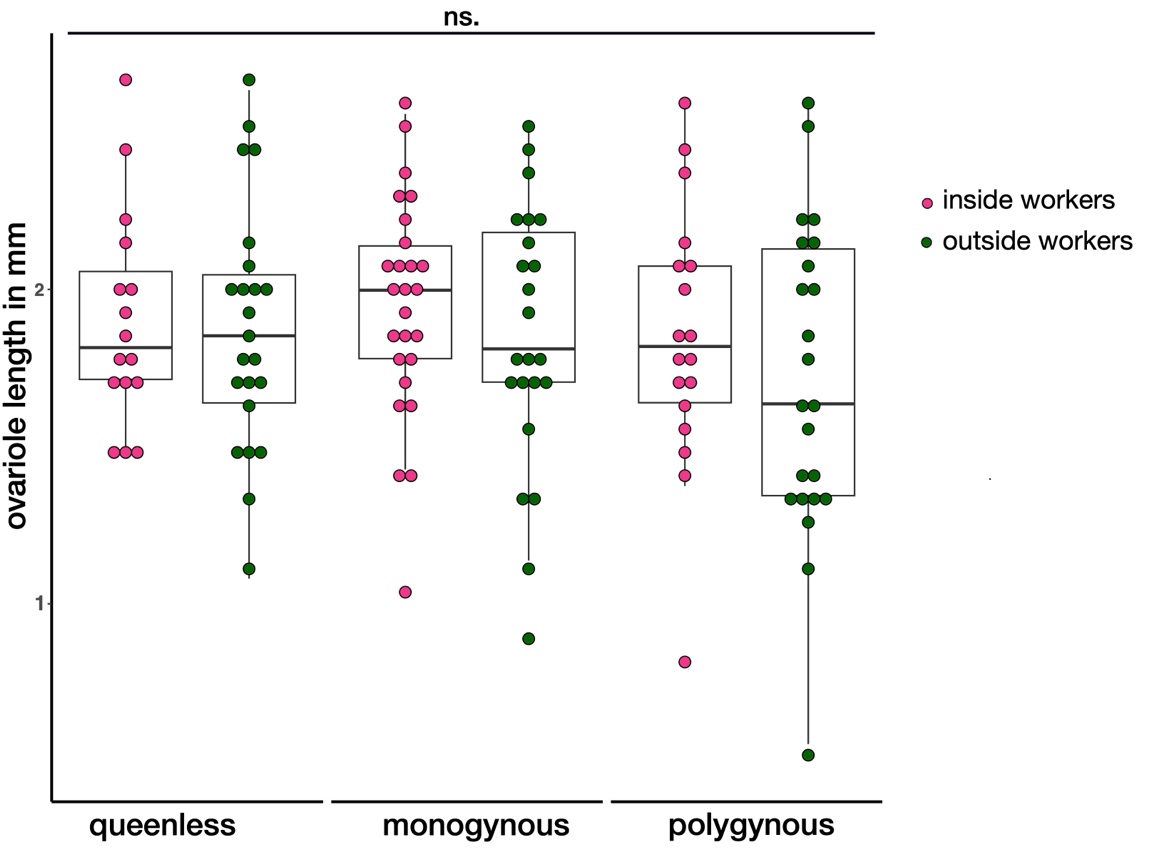


Figure S 2: Mean ovariole length within inside and outside workers of the three queen treatments (queenless, monogynous, polygynous). Inside and outside workers had similarly long ovarioles, independent to queen number (LMER_queen number_: X^2^= 1.17, p= 0.57; LMER_worker location_: X^2^ = 2.75, p= 0.10).


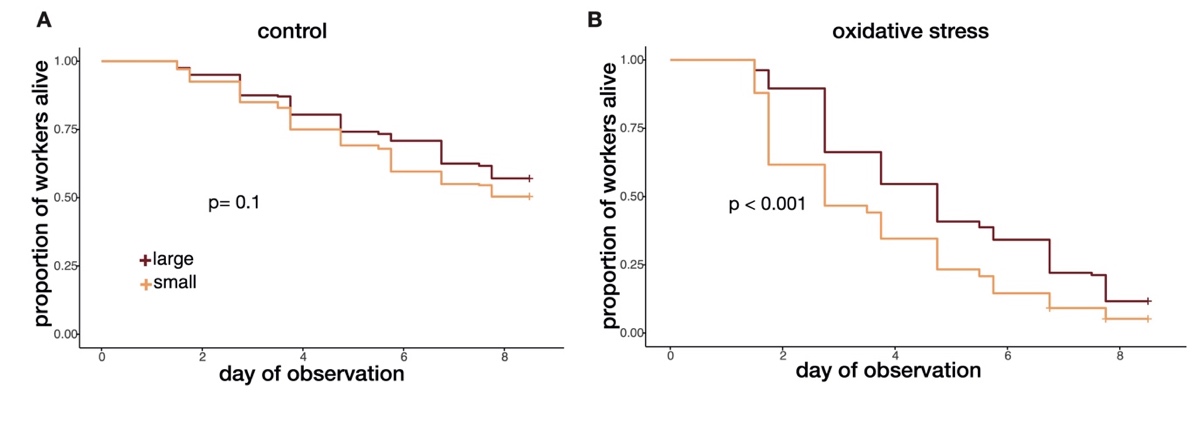


Figure S 3: Worker survival dependent to worker size within the oxidative stress experiment. A) Large (dark red line) and small (orange line) workers within the control treatment survived similarly long (X^2^= 2.69, p= 0.1). B) Large workers did survive better than small workers when subjected to paraquat- induced oxidative stress (X^2^= 46.11, p< 0.001).


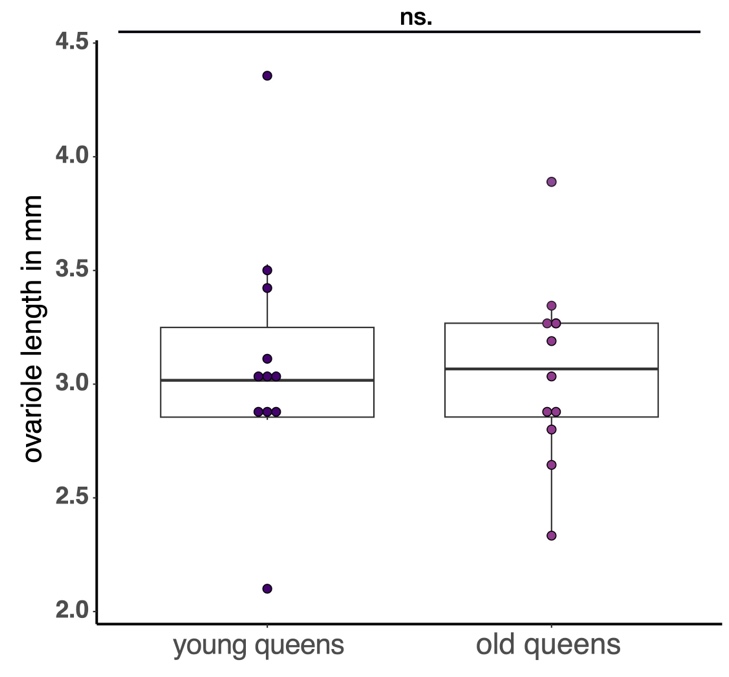


Figure S 4: Mean ovariole length of young and old Tapinoma magnum queens. Young and old queens had similar long ovarioles (LMER: X^2^= 0.02, p= 0.811).

Table S 1: Significantly enriched GO Terms (molecular function) in the list of genes that were overexpressed in inside workers compared to outside workers including the respective p-values obtained from the Fisher’s exact test.

| **GO-ID** | **Term** | **Annotated** | **Significant** | **Expected** | **Fisher’s exact test p-value** |
| --- | --- | --- | --- | --- | --- |
| **GO:0016705** | oxidoreductase activity, acting on paired donors, with incorporation or reduction of molecular oxygen | 368 | 29 | 6.79 | 2.1E-09 |
| **GO:0005506** | iron ion binding | 377 | 25 | 6.96 | 3.2E-08 |
| **GO:0004497** | monooxygenase activity | 323 | 21 | 5.96 | 4.8E-07 |
| **GO:0005515** | protein binding | 4385 | 128 | 80.93 | 5.1E-07 |
| **GO:0003723** | RNA binding | 1254 | 49 | 23.14 | 9.9E-06 |
| **GO:0005509** | calcium ion binding | 496 | 22 | 9.15 | 0.00016 |
| **GO:0020037** | heme binding | 437 | 20 | 8.07 | 0.00021 |
| **GO:0008017** | microtubule binding | 131 | 9 | 2.42 | 0.00075 |
| **GO:0000981** | DNA-binding transcription factor activity, RNA polymerase II-specific | 179 | 8 | 3.3 | 0.03658 |
| **GO:0005085** | guanyl-nucleotide exchange factor activity | 139 | 6 | 2.57 | 0.04448 |

Table S 2: Significantly enriched GO Terms (molecular function) in the list of genes that were overexpressed in outside workers compared to inside workers including the respective p-values obtained from the Fisher’s exact test.

| **GO.ID** | **Term** | **Annotated** | **Significant** | **Expected** | **Fisher’s exact test p-value** |
| --- | --- | --- | --- | --- | --- |
| **GO:0005515** | protein binding | 4385 | 209 | 123.95 | 1.3E-19 |
| **GO:0020037** | heme binding | 437 | 37 | 12.35 | 3.7E-09 |
| **GO:0004497** | monooxygenase activity | 323 | 31 | 9.13 | 4.3E-09 |
| **GO:0005506** | iron ion binding | 377 | 33 | 10.66 | 4.5E-09 |
| **GO:0016705** | oxidoreductase activity, acting on paired donors, with incorporation or reduction of molecular oxygen | 368 | 32 | 10.4 | 9.2E-09 |
| **GO:0005509** | calcium ion binding | 496 | 30 | 14.02 | 9E-05 |
| **GO:0004181** | metallocarboxypeptidase activity | 36 | 6 | 1.02 | 0.00047 |
| **GO:0004842** | ubiquitin-protein transferase activity | 170 | 13 | 4.81 | 0.00122 |
| **GO:0004252** | serine-type endopeptidase activity | 314 | 19 | 8.88 | 0.00166 |
| **GO:0008270** | zinc ion binding | 975 | 44 | 27.56 | 0.00167 |
| **GO:0005085** | guanyl-nucleotide exchange factor activi... | 139 | 10 | 3.93 | 0.00624 |
| **GO:0004674** | protein serine/threonine kinase activity | 158 | 11 | 4.47 | 0.01036 |
| **GO:0004222** | metalloendopeptidase activity | 207 | 12 | 5.85 | 0.01514 |
| **GO:0031267** | small GTPase binding | 72 | 6 | 2.04 | 0.01614 |
| **GO:0140359** | ABC-type transporter activity | 145 | 10 | 4.1 | 0.01809 |
| **GO:0043169** | cation binding | 2690 | 136 | 76.04 | 0.01945 |
| **GO:0016747** | acyltransferase activity, transferring g... | 446 | 15 | 12.61 | 0.02366 |
| **GO:0016298** | lipase activity | 111 | 9 | 3.14 | 0.03566 |
| **GO:0050660** | flavin adenine dinucleotide binding | 311 | 13 | 8.79 | 0.04072 |
